# Integrated physical and mental management for China’s elderly: insights from Long-gang District, Shenzhen

**DOI:** 10.3389/fragi.2024.1361098

**Published:** 2024-02-06

**Authors:** Xun qiang Wang, Ce ce Yang, Xi ling Sun

**Affiliations:** ^1^ Longgang Center for Chroinc Disease Control, Shenzhen, China; ^2^ Shanghai Huangpu Mental Health Center, Shanghai, Shanghai, China

**Keywords:** elderly, aging, physical health, mental health, public health, health management, integrated care

## Abstract

China is in a period of rapid population aging. The total population of the elderly aged 60 and above in mainland China was 264 million in 2020, and is the country with the largest elderly population in the world, which is home to 1/5 of the world’s older people. The urgency of actively coping with the aging population has never been greater, and China has raised it to the height of national strategy. To this end, China has issued several plans and projects on aging work. Many of them include multiple overlapping components. The management of physical illness and mental illness in the elderly is over-differentiated and segmented. However, it is common for older adults with complex health problems. The body and mind are inherently integrated and interact with each other, and should not be separated. There is an urgent need for integrated healthcare services for the physical and mental health of the elderly population. The national basic public health services play an important role in early detection and awareness of health problems for the elderly in community health services. This paper introduces the elderly health management services, one of the national basic public health projects, and the psychological care project for the elderly in Shenzhen, Guangdong Province, China. Taking Long-gang District’s exploration of the joint management of physical and mental health of the elderly as an example, this review discusses the difficulties of the elderly health work, and the feasibility of integrating the elderly mental health and physical health in medical care. We outlook to build an integrated platform for physical and mental health of the elderly in China. Focus on the needs of older population, strengthen community health services, build a integrative team, fully publicize and improve health literacy of the elderly, link up and down and work together, improve coordination between providers of medical care and social services. It is of great significance to construct a strong public health system for the elderly and promote the realization of the grand goal of Healthy China.

## Introduction

Since 2000, China has stepped into an aging society. China is in a period of rapid population aging, and is the country with the largest elderly population in the world, which is home to 1/5 of the world’s older people. The results of the Seventh National Population Census in 2020 showed the total population of the elderly aged 60 and above in mainland China was 264 million, accounting for 18.7% of the total population. The proportion of the population aged 65 and above in China has reached 13.50%, and the degree of population aging is higher than the world average (9.3% of the population aged 65 and above), but lower than the average level in developed countries (19.3% of the population aged 65 and above) (Finance.People, 2023). As in the rest of the world, declining fertility and rising life expectancy have both contributed to population ageing in China. Not like other countries, China is facing challenges of large scale of older population, significantly accelerating in the aging process, significant difference in the level of aging between urban and rural areas, getting old before getting rich, the pressure of public service supply.

The health of the elderly is generally less than perfect (Sowa et al., 2015). The older often suffer from multiple diseases, and their functional capacity is frequently limited (Marengoni et al., 2011), and the decline of cognitive, physical functions as well as nutrition, psychological and other health problems associated with aging are increasingly prominent. The prevalence of chronic diseases in the elderly is 2.3–3.2 times that of the whole population. About 65% of the elderly in China suffer from two or more diseases of different degrees at the same time. With the growth of age, the number of elderly people with multiple diseases is increasing (Singh et al., 2004; Sikka et al., 2015; QI et al., 2023). The China Mental Health Survey published in Lancet Psychiatry 2019 showed the weighted prevalence of dementia in Chinese elderly aged 65 years or older in 2013 was 5.6%, and other mental disorders (excluding dementia) was 4.9% (Huang et al., 2019). With a history of decades of illness, the etiology of Alzheimer’s disease is unknown. Dementia and other non-communicable chronic diseases share several risk factors, such as hypertension, hyperglycemia, unhealthful lifestyle, and unhealthful behaviors. Multi-domain interventions that target these spots at the same time might be needed for effective prevention (Jiang et al., 2013; Xu et al., 2015; Kivipelto et al., 2018; Silva et al., 2019; Zhang et al., 2021; Zhang et al., 2022).

Most of countries in the worldwide face major challenges to manage the elderly health. In the period of economic underdevelopment, facing the challenge of population aging, the most basic countermeasures are to realize healthy aging, improve the community elderly care service system, and strengthen healthcare and psychological support for the elderly. The urgency of actively coping with the aging population has never been greater, and China has raised it to the height of national strategy. Implementing Healthy China and Actively Responding to Population Aging are China’s Two National Strategies.

To this end, China has issued several plans and projects on aging work, however, many of them include multiple overlapping components. China included elderly health services for the elderly over 65 years of age nationwide into the basic public health program in 2009. The General Office of the State Council of China issued a notice on the formulation and implementation of Opinions on elderly care service projects in 2017, provided free health management services including physical examination for the elderly aged 65 and above every year (Office of the State Council, 2023). Strengthening the Work on Aging in the New Era was released in 2021. Psychological care for the elderly, oral health, nutritional improvement, and dementia prevention and treatment are four actions to be implemented in China. The leading departments of these projects on the elderly health management in China, are not one, but always many, which lead to many problems and difficulties.

The management of physical illness and mental illness in the elderly is over-differentiated and segmented. However, it is common for older adults with complex health problems (Meinow et al., 2015; Ho et al., 2017). The body and mind are inherently integrated and interact with each other, the progress of mental health will affect physical health, and should not be separated (Saha et al., 2013; Doherty and Gaughran, 2014; Tegethoff et al., 2015; De Hert et al., 2018). The distinction between “mental” disorders and “physical” disorders is a reductionistic anachronism of mind/body dualism. It is impossible to identify any characteristic features of either the symptomatology or the aetiology in linguistic distinction between so-called mental illnesses and so-called physical illnesses (Kendell, 2001). In the elderly, a new onset of psychosis is usually associated with an organic cause (Singh et al., 2004). Sick elderly adults often suffer from both so-called physical and mental illnesses, making it even more important for them to manage their physical and mental health together. The elderly adults with complex health problems often need a variety of services. Medical institutions can regularly conduct physical examinations and psychological screening for the elderly at the same time, to evaluate their physical and psychological condition. Based on the evaluation results, provide targeted intervention measures.

Community is the basic environment for the elderly and the main place for the implementation of elderly healthcare. Primary healthcare is the gatekeeper. Primary medical and health institutions are the dual network base of basic medical services and public health services. In this paper, we introduce the elderly health management services, one of the national basic public health projects, and the psychological care project for the elderly in Shenzhen, Guangdong Province, China. Taking Long-gang District’s exploration of the joint management of physical and mental health of the elderly as an example, this paper discusses the difficulties of the elderly health work, and the feasibility of integrating the elderly mental health and physical health in medical care. We outlook to build an integrated platform for physical and mental health of the elderly in China.

## Standards for elderly health management services in national basic public health services

The national basic public health service project is an important part of the construction of China’s public health system, which aims to promote the gradual equalization of basic public health services. Since 2009, China has included elderly health services for the elderly over 65 years of age nationwide into the basic public health program. The goal of the project is to improve the quality of life and health of the elderly population. The services need provide health management for the elderly once a year, including lifestyle and health assessment, physical examination, auxiliary examination, and health guidance. By conducting consultations and self-assessment of the health status of the elderly, to evaluate their basic health status, physical exercise, diet, smoking, alcohol consumption, common symptoms of chronic diseases, past medical history, treatment, current medication, and self-care abilities. Perform routine physical examination, including body temperature, pulse, breathing, blood pressure, height, weight, waist circumference, skin, superficial lymph nodes, lungs, heart, abdomen, etc., and rough testing of oral cavity, vision, hearing, and exercise functions, etc. Auxiliary examination include blood routine, urine routine, liver function, renal function, fasting blood glucose, blood lipids, electrocardiogram, and abdominal B ultrasound examination. Provide corresponding health guidance such as healthcare and disease prevention based on the evaluation results.

Primary medical and health institutions, include township hospitals, village clinics and community health service centers (stations), which provide free and voluntary basic public health services to elderly residents. The superior department such as local disease control and prevention, health supervision and other professional public health institutions need to supervise the completion of this work and provide technical guidance and relevant skills training. Government departments provide financial support.

### Work specification for Shenzhen elderly health management and integrated medical care services

Local health authorities at all levels can improve and perfect the national basic public health service mode according to the basic requirements of the national standards and the local actual situation. In 2019, the combination of medical care and elderly care was included in the national basic public health service. Shenzhen Health Commission linked the elderly health management with the combination of medical care and elderly care. Since then, the elderly health management and integrated medical care services had been carried out for the elderly aged 65 and above in Shenzhen. The goal is to improve the quality of life and health of the elderly, and to improve the quality of life of the disabled elderly.

Integrated medical and elderly care services include blood pressure measurement, peripheral blood glucose testing, rehabilitation guidance, nursing skill guidance, health consultation, and nutrition improvement guidance for individuals aged 65 and above who live at home. These services are provided twice a year by primary care institutions based on the results of previous health examinations of the elderly. Provide door-to-door health assessment and health services to the elderly with advanced age, disability and mobility problems. These services include nursing guidance and psychological support at least once a year.

### Shenzhen psychological care project for the elderly

Shenzhen decided to carry out the psychological care project for the elderly in 2022. The work goal is to improve the mental health work system, standardize the service process of the elderly. To establish mental health management model of elderly population, the project includes initial screening and reevaluation, diagnosis and treatment of mental illness, and follow-up intervention in community health service institutions. The project uses relevant, extensively used, feasible, and valid scales for assessment of mood and cognition of the elderly population in Shenzhen. Cognitive function assessment by using Ascertain Dementia 8 (AD8) (Chin et al., 2013), emotional state by using The Patient Health Questionnaire-2(PHQ-2) (Kroenke and Williams, 2003) and by the 7-item Generalized Anxiety Disorder Questionnaire (GAD-7) (Spitzer et al., 2006) is the initial screening for all aged residents in Shenzhen. If the assessment results of AD8 ≥ 2 points, or PHQ-2 ≥ 3 points, or GAD7 ≥ 5 points, it will be judged as positive in the preliminary screening. The medical staff of basic medical and health institutions will use the Geriatric Depression Screening Scale (GDS-15) (Burke et al., 1991) and the Mini-Mental State Examination (MMSE) (Folstein and McHugh, 1975; Li et al., 2016) to then recheck the elderly with positive emotional status or cognitive function assessment. If the result of reevaluation is GDS-15 ≥ 9 points, or GAD-7 ≥10 points (follow the preliminary screening score), or MMSE score less than the cut-off value divided by years of education (the cut-off points <17 for illiterate, ≤20 for individuals value with 1–6 years of education, and ≤24 for individuals with 7 or more years of education), it will be judged as emotionally or cognitively high-risk of elderly individuals.

The elderly with high risk should be reported in time and issued a referral to medical institutions or hospitals that have departments for mental or cognitive disorders for further diagnosis and treatment. Basic medical and health institutions need to regularly report to district level of prevention and control center, which will control and supervise the quality of relevant data submitted. The elderly adults are divided into three groups, according to the preliminary screening and reevaluation results, respectively implemented different interventions. For example, provide mental health education for the whole elderly population, and carry out follow-up management and self management group intervention for the elderly with abnormal screening results.

The psychological care project for the elderly was conducted in 789 community health centers. The number of permanent elderly residents aged 65 or above in Shenzhen is 604967 in 2023.516799 permanent residents completed preliminary screening in 2023.10571 elderly people were positive screening results in emotion or cognition. 9,829 completed revaluation in emotion and cognition. 2,144 were emotionally or cognitively high-risk. Follow-up and referral were completed in 87.03% and 82.37% of high-risk elderly, respectively. The mental health popularization such as distributing popular science materials, carrying out lectures and free consultation was supplied to all the elderly. Self-health management group activities, such as to build cognitive reserves, engage in cognitive retraining, mood management, and create social networks, provide social support and social participation, were carried out for high-risk elderly.

## Problems and difficulties

To ensure the smooth implementation of the above projects, the government needs to provide financial support. However, because of the financial tightening and other reasons, it is extremely difficult to implement these work independently. There is a lack of professional institutions for promoting elderly health, and the prevention and control of key diseases in the elderly are weak in China. The development of elderly medical and health institutions is insufficient, and there is a serious shortage of rehabilitation hospitals, nursing homes, and hospice centers. The foundation of elderly health services such as comprehensive assessment of the elderly, management of elderly syndrome, and multidisciplinary diagnosis and treatment is weak.

Compared with other specialties, geriatric talents are in short supply. The number of geriatricians in China is still less than one-10th of the actual demand, far from meeting the needs of 264 million elderly people. The development of geriatric medicine and related disciplines is lagging behind. Compared with other disciplines, psychiatry has fewer professionals. According to the statistics of the National Health Commission, by the end of 2021, the number of psychiatrists in China is 64000, accounting for only 1.49% of the national total (4.287 million). There is an extreme shortage of geriatric psychiatrists. The existing geriatric doctors are mostly composed of different specialties, with solid specialized knowledge but insufficient general knowledge, rich experience in disease diagnosis and treatment, but insufficient experience in long-term care management.

There is a shortage of elderly health service personnel, especially grassroots personnel. Most medical staff of basic medical and health institutions in Shenzhen City, China, are general practitioners. In recent years, although a few of them have participated in the post transfer training of psychiatrists, which can add to the scope of psychiatric practice, they still lack relevant skills and professional abilities. It is difficult for them in the diagnosis and treatment of mental diseases in the elderly. It is difficult for them to manage the elderly health independently. The ability to provide home care and care services for the elderly and disabled elderly urgently needs to be strengthened.

It is difficult to reestablish a team of elderly psychological care workers. After more than 4 years of implementation, the elderly health management and integrated medical care services have formed a team of elderly physical health management talents. Basic medical and health institutions are the main body to undertake the elderly service task. COVID-19 pandemic had lasted for 3 years, and the task of epidemic prevention was heavy for the medical staff in basic medical and health institutions. The new psychological care project for the elderly had once again added new service tasks to the grass-roots medical and health service personnel.

If a project involves multiple departments, it is easy to kick the ball. It needs a department to lead and a integrated team to participate. With the financial tightening of financial departments at all levels in the economic downturn and the shortage of geriatric mental health professionals, it is impractical to establish another senior mental health management team. Our survey found that most grassroots medical institutions are the same group of staff to do both the elderly health management and integrated medical care services, and elderly psychological care projects. However, the leading departments involve different departments and almost no communication. There were duplicate submission materials and waste of resources.

## Outlook

The work content of the elderly health management and integrated medical care services involves psychological support and other psychological services, which are overlapping with the elderly psychological care project. It is common for the elderly to suffer from both physical and psychological diseases. It is almost impossible to completely distinguish between so-called physical and mental diseases (Kendell, 2001). Therefore, it is necessary and feasible to manage the body and mind of the elderly at the same time. The top-level design should integrate the elderly health management and integrated medical care services with the elderly psychological care project.

Shenzhen Long-gang District explores the integration of the elderly psychological management project with the elderly health management and integrated medical care services, so as to achieve the management of body and mind together. The superior health administrative department integrates the medical, care and social resources in Long-gang District, establish elderly health guidance center, guide primary medical institutions, hospitals, rehabilitation centers and nursing homes, comprehensively promote the health work of the elderly. These work are provided financial support in the budget for basic public health services. A unified electronic health information system was established and enable streamlined coordination among providers at different tiers, and a standardized referral system would be put in place to integrate the provision of care across primary medical center, hospitals, rehabilitation centers, and health administrative department (Chen et al., 2022). Primary medical staff can not only do physical assessment, intrinsic abilities, but also psychological assessment, and give physical and psychological nursing guidance, rehabilitation, health promotion and prevention, and health management and maintenance for chronic conditions. Primary medical staff identify the need for further diagnosis and treatment, and make recommendations for referral. Hospitals, rehabilitation centers and nursing homes provide medical services, rehabilitation and nursing services by tiered health delivery system. The elderly health guidance center establishes integrated team, provides professional training, technical guidance and professional support for the basic medical and health institutions, establish two-way referral mechanism and provides further diagnosis and treatment services for the elderly patients referred. For example, in terms of physical and psychological evaluation of the elderly, primary medical staff are ongoing trained and supervised by senior professionals of hospitals.

In 2023, more than 85% elderly people were screened and assessed for physical and cognitive and emotional functions in Long-gang District. Based on the results, the elderly health management staff provided further assessment, referral, diagnosis and treatment, intervention and nursing. Promote health literacy of older adults, to improve awareness and understanding of risk factors for and health practices related to physical and mental health.

Although the integration of physical and mental management still faces problems and difficulties, such as multi-department collaboration, insufficient information platform intelligence, weak ability of professionals, this innovative measure can optimize the working mechanism, reduce labor costs, duplication of work, and waste of fund (Schwarz et al., 2022). We call for establishing a working system for the simultaneous management of physical and mental health of the elderly, and publicizing relevant policies, strengthening the publicity of importance of healthcare service integration of physical and mental health in aging, increasing service delivery evaluation and needs assessment of older people, constructing a self-recording platform integrated with electronic health information system, providing health education, and funding increases (Karako et al., 2019; He and Tang, 2021). To solve the shortage of geriatric medicinal providers, the government should provide short-term or long-term training to geriatricians, and geriatricians transform the geriatrics workforce to staff who do not specialize in the elderly care in their respective regions, to rise the number as well as increase the capability of service in near future. It is also necessary to increase the open dialogue between medical workers, sociologists, economists, social workers, and other gerontologists, strengthen the research on the elderly, learn advanced knowledge in gerontology from other countries (Davey et al., 2014), and provide evidence-based evidence for better achieving successful aging (Frost et al., 2020).

With extending of the average of life span, China has stepped into aging society, with more than 200 million population of the elderly more than 65 years old. The health problem of the elderly has increased the burden on the family and society, which is also an important public health problem. It can be predicted that difficulties of one kind or another will inevitably occur in the process of healthcare integration. However, if healthcare services are provided based on the needs of the elderly, it is believed that the elderly health team can form a joint force and play a better role in healthcare.

The best action to protect the health of the elderly is in the community and health promotion and education in advance. The best way to manage elderly health is to prevent it. Safeguarding the health of the elderly requires joint action by the whole society. In addition to the medical system, resources such as nursing homes, elderly care institutions and community elderly activity centers need to be integrated and coordinated by government. The division of responsibilities of relevant departments mentioned above should been written into the project of combination of physical and mental health and medical care for the elderly. We once again call on top designers to integrate resources, establish integrative team, and fully publicize and improve the physical and mental health literacy of the elderly. It is of great significance to construct a strong public health system for the elderly and promote the realization of the grand goal of Healthy China.
